# Ontogenetic variations and structural adjustments in mammals evolving prolonged to continuous dental growth

**DOI:** 10.1098/rsos.170494

**Published:** 2017-07-26

**Authors:** Helder Gomes Rodrigues, Rémi Lefebvre, Marcos Fernández-Monescillo, Bernardino Mamani Quispe, Guillaume Billet

**Affiliations:** 1 Centre de Recherche sur la Paléobiodiversité et les Paléoenvironnements (CR2P), UMR CNRS 7207, CP38, Muséum national d'Histoire naturelle, Univ Paris 6, 8 rue Buffon, 75005 Paris, France; 2 Mécanismes adaptatifs et évolution (MECADEV), UMR 7179, CNRS, Funevol team, Muséum National d'Histoire Naturelle, 55 rue Buffon, Bat. Anatomie Comparée, CP 55, 75005, Paris Cedex 5, France; 3 Instituto Argentino de Nivología, Glaciología y Ciencias Ambientales (IANIGLA), CCT–CONICET–Mendoza, Avda. Ruiz Leal s/n, Parque Gral, San Martín 5500, Mendoza, Argentina; 4 Departamento de Paleontología, Museo Nacional de Historia Natural, Calle 26 s/n, Cota Cota, La Paz, Bolivia

**Keywords:** dental complexity, inhibitory cascade model, dental mesial drift, notoungulates, gundies

## Abstract

Studying dental ontogeny in mammals can provide valuable insight on the evolution of their masticatory apparatus and their related adaptations. The multiple acquisitions of a prolonged to continuous growth of teeth in herbivorous mammals in response to high abrasion represent an intensively investigated issue. However, the ontogenetic and architectural patterns associated with these repeated dental innovations remain poorly known. Here, we focused on two case studies corresponding to distant mammalian clades, the extinct Mesotheriidae (Notoungulata), which shared some striking dental features with the extant Ctenodactylidae (Rodentia). We studied the impact of prolonged to continuous growth of molars on their occlusal complexity, their relative size and their dynamics in the jaw. We found that variations of occlusal complexity patterns are the result of paedomorphic or peramorphic heterochronic processes impacting dental crown. We showed that variations in both upper and lower molar proportions generally follow the inhibitory developmental cascade model. In that context, prolonged dental growth implies transitory adjustments due to wear, and also involves dental migration and loss when combined with molar lengthening. Interestingly, these features may be present in many mammals having prolonged dental growth, and emphasize the crucial need of considering these aspects in future evolutionary and developmental studies.

## 1. Introduction

Studies of dental development in mammals have long been interested in the growth, modes of replacement and structural morphology of teeth, in order to better understand their evolution [1–6]. The prolonged to continuous growth of teeth is a striking and repeated innovation of many herbivorous mammals, which allow them to have a more durable function to compensate their limited dental replacement compared with other vertebrates [7–9]. Recently, the underlying developmental mechanisms and associated gene signalling pathways involved in the prolonged growth of teeth gained more attention in order to better understand how these innovations convergently appeared and evolved in different clades of mammals [10–12]. However, modifications in the occlusal designs and relative size variations within the tooth row associated with the setting of prolonged to continuous dental growth remain poorly known, as well as inherent structural and mechanical constraints, and resulting adjustments within the masticatory apparatus (e.g. [13]). The study of some of these aspects in relation to the known developmental mechanisms governing prolonged to continuous dental growth could be of high interest to understand how they contribute to both homeostasis and evolution of an efficient dentition in extinct and extant mammals.

Among placental mammals, high-crowned cheek teeth have been reported to have evolved first in South American native ungulates (i.e. notoungulates) and in diverse families of rodents, lagomorphs and xenarthrans during the Eocene [14–17]. Among them, the extinct clade of South American notoungulates is a suitable model to study the ontogenetic variations and structural adjustments associated with increasing crown height, inasmuch as they acquired prolonged to ever-growing dentitions several times during their evolutionary history [18,19]. Debates on the causes of high dental wear experienced by these notoungulates recently favoured the abundance of dust and grit supposedly deposited on ingested plants due to intense volcanism and increasing aridity in South America by the Late Eocene onwards (e.g. [17,20]). The convergent increase of crown height in association with faster molar eruption recently evidenced in four main clades of notoungulates may have also been influenced by these environmental pressures, and facilitated by inherent ontogenetic and structural adjustments within their masticatory apparatus, which remained to be evidenced [19].

**Figure 1.**
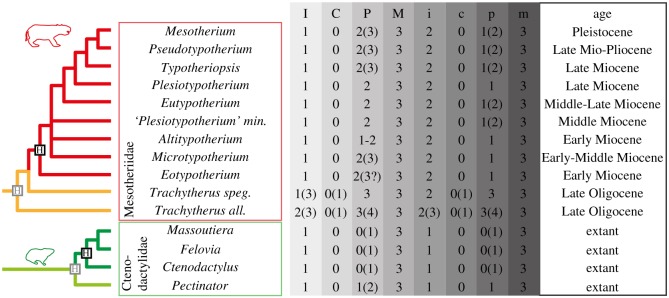
Phylogeny of the Mesotheriidae and the Ctenodactylidae [21,25] with data on the dentition (crown height, dental formula) and age. Grey H: Hypsodont, Black H: Hypselodont. Names of ‘*Plesiotypotherium*’ *minus*, *Trachytherus spegazzinianus* and *Trachytherus alloxus* were shortened. Maximal number of teeth in brackets.

Among the structural features frequently associated with prolonged growth in notoungulates, some have raised questions on the evolution of their dental formula and dental ontogeny (e.g. [9,19,21,22]), but they remain poorly studied. These features are the strong reduction of the dentition, the increase of dental proportions during early ontogenetic stages, and the strong enamel infolding of the occlusal pattern, which is uncommon in other ungulates. It is noteworthy that, among these notoungulates, the Mesotheriidae show all these features and displayed striking convergences with other mammals, in having a reduced dentition combined with a diprotodont masticatory apparatus (i.e. two front teeth combined with a large diastema separating them from the cheek teeth; [23,24]), like rodents and some marsupials. Interestingly, the combination of increasing dental specializations seen in Mesotheriidae, such as prolonged growth associated with strong modifications of the occlusal pattern and teeth loss during life, are similar to rodents, such as gundies (figure 1). Ctenodactylidae, or African gundies, currently encompass four genera which evolved in open and arid environments [26], as probably did most of mesotheriids in South America (known only at high latitudes or high altitudes; e.g. [27]).

Here, we choose to investigate the variations in occlusal complexity, spatial arrangement and dynamics of molars within the jaw in Mesotheriidae. These data are compared with those obtained on Ctenodactylidae, for which data about dental dynamics are already known, because migration of molars has been documented in this clade [28]. The interest of studying these phylogenetically distant but partly resembling clades of mammals relies on the fact that both groups encompass taxa with high-crowned (hypsodont) and ever-growing dentitions (hypselodont), which are well-documented by ontogenetic series. The scrutiny of both families allows a detailed preliminary assessment of some major ontogenetic variations and structural adjustments potentially associated with the evolution of prolonged to continuous growth of teeth. More generally, this study also provides an original comparison of the ontogenetic and evolutionary trajectories of mammals displaying convergent features, but different sizes and inherited dental patterns, and close adaptive strategies related to increasing dental wear.

## 2. Material and methods

### 2.1 Dentitions of Mesotheriidae and Ctenodactylidae

Nine extinct genera of relatively small to medium-sized Mesotheriidae (Notoungulata) compared with four extant genera of small-sized Ctenodactylidae (Rodentia) were considered in this study (figure 1). Hypsodonty indices were not considered in this study, because they were poorly relevant for hypselodont taxa and showed quite similar values between hypsodont and hypselodont taxa (around 2) in Mesotheriidae [24]. Data concerning lower and upper dentitions (35 lower and 58 upper dentitions for Mesotheriidae; 57 lower and 57 upper dentitions for Ctenodatylidae) were obtained based on juvenile and adult specimens housed in the MNHN (Paris, France) for both the Mesotheriidae and Ctenodactylidae, and from the MACN (Buenos Aires, Argentina), the MLP (La Plata, Argentina), the UNPSJB (Comodoro Rivadavia, Argentina), the MNHN-Bol (La Paz, Bolivia) regarding some Mesotheriidae, and also from the literature (e.g. [21,29], but see the electronic supplementary material, table S1 for more details). Our analyses focused on molars, which are the predominant functional cheek teeth in these clades, due to the loss of some premolars. Moreover, molars were shown to have high morphological and developmental integrations in mammals [4,30,31].

### 2.2. Dental measurements and complexity index

**Figure 2.**
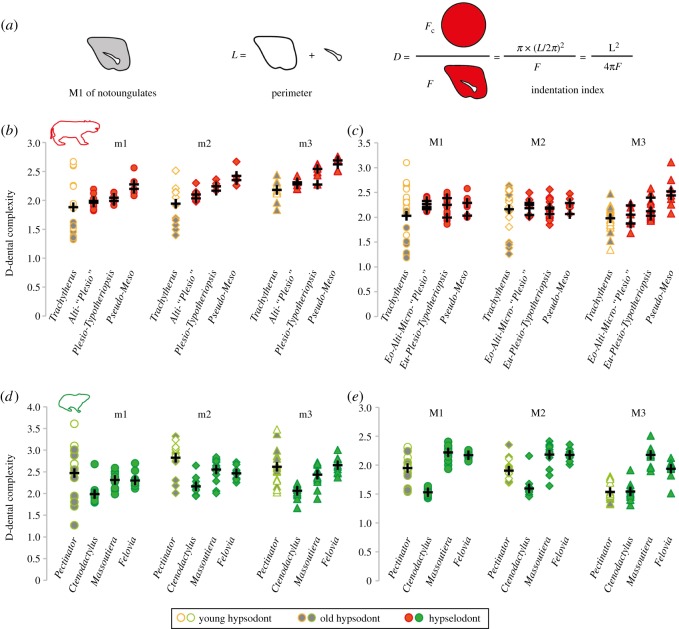
Graphs showing dental complexity variations in hypsodont and hypselodont Ctenodactylidae and Mesotheriidae. (*a*) Schematic representation of the calculation of the indentation index (D) on a worn M1 of *Trachytherus*. This parameter is calculated as a ratio that corresponds to the calculated circular area (*F*_c_) whose perimeter (*L*) corresponds to the total measured length of the enamel bands divided by the measured area of the occlusal surface (*F*, modified from [32]). (*b*,*c*) Lower and upper dental complexity in Mesotheriidae classified by age and phylogenetical affinities. (*d*,*e*) Lower and upper dental complexity in Ctenodactylidae, by genus.

**Figure 3.**
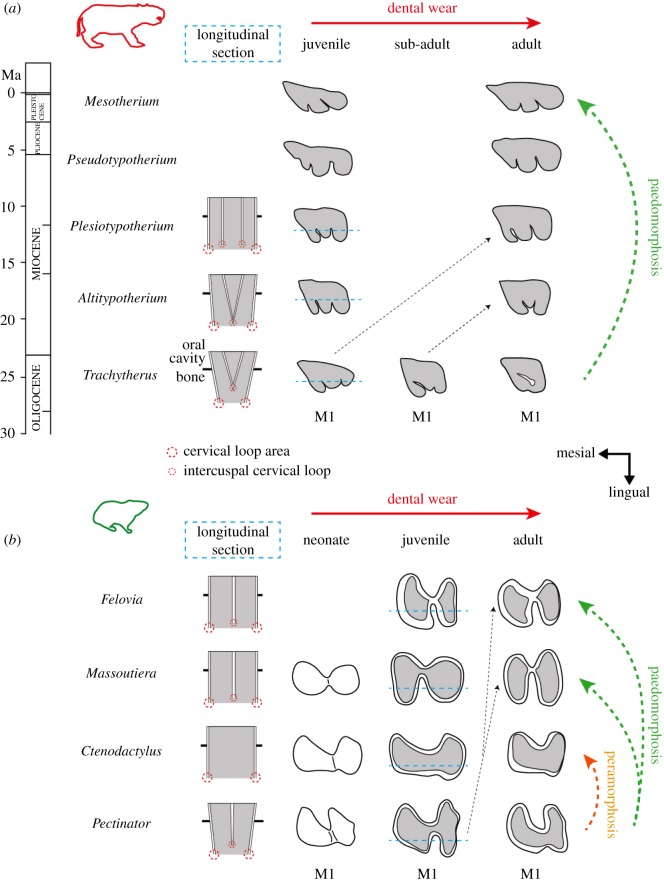
Dental diagrams of occlusal surface and longitudinal section of molars showing potential ontogenetic changes occurring during (*a*) mesotheriid evolution and (*b*) between ctenodactylids. Black dotted arrows represent corresponding occlusal morphotypes, between different species at different ontogenetic stages. Diagrams of longitudinal sections show putative modes of molar development from hypsodont to hypselodont patterns with depiction of cervical loop areas corresponding to stem cell niches.

Dentitions were digitized with a camera (Canon SD60 and 550D) in a standardized manner, and then outlines of enamel occlusal surface, including fossae, of upper and lower molars were measured using ImageJ. All available wear stages of molars were considered for complexity measurements, as long as a given specimen preserved at least the mesialmost molar. For *Trachytherus*, the relative wear stages of the selected specimens were determined based on Billet *et al*. [27]. For both Mesotheriinae (i.e. Mesotheriidae without *Trachytherus*) and Ctenodactylidae, adults were arbitrarily defined on the basis of a well-erupted and worn permanent dentition (including M3, and permanent premolars when present). Areas and perimeters were then calculated, as well as the indentation index (D) following Schmidt-Kittler [32,33], a method which was also recently used in ungulates [34]. The indentation index corresponds to the circular area, whose perimeter equals the total measured length of the enamel bands divided by the measured area of the occlusal surface (figure 2*a*; [33]). This index allows quantifying the complexity of the occlusal surface and tracing its evolution along ontogenetic series in both hypsodont and hypselodont Mesotheriidae (classified by age and phylogenetical affinities) and Ctenodactylidae (classified by genus; figures 2 and 3). Kendall's τ was performed with PAST [35] to test the correlation between dental size and dental complexity for each molar (electronic supplementary material, table S2), and for each mode of growth (i.e. hypsodont and hypselodont), as measured in extinct and extant equids for which complexity is negatively correlated with occlusal area [36]. The effect of phylogeny was not considered in these analyses partly because our samples are largely focused on the intraspecific (or intragroup) variations of these features rather than on their interspecific variations (electronic supplementary material, table S1).

### 2.3. Testing the inhibitory developmental cascade model

**Figure 4.**
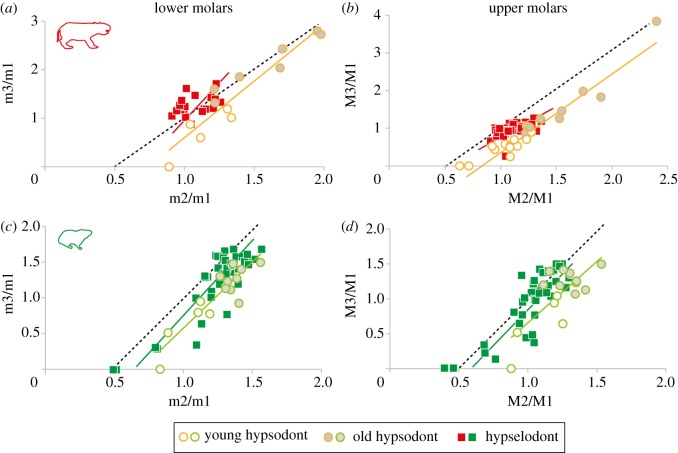
Graphs showing variations in molar proportions ratio compared to IC model in hypsodont and hypselodont (*a*,*b*) Mesotheriidae and (*c*,*d*) Ctenodactylidae. The dashed lines correspond to the regression of the experimental IC model, while solid lines correspond to regression lines of hypsodont and hypselodont specimens.

Relative size arrangements of molars were assessed using the inhibitory developmental cascade index (i.e. IC model; [4]). The IC model illustrates the constraints occurring during dental formation, especially the putative inhibitory role of first developing molars on subsequent teeth, which may mainly drive the variation of molar proportions observed in mammals [37]. The matching of mesotheriid and ctenodactylid molars with the IC model was analysed through the investigation of molars ratio (i.e. M2/M1 versus M3/M1), following Kavanagh *et al*. [4], who measured it for lower molars. We realized this investigation for the first time in the context of prolonged dental growth to evaluate the evolution of molar size ratio at different wear stages, and for both lower and upper molars to test whether both sets follow similar ontogenetic trajectories (figure 4). The full area of a given molar was considered in this analysis (i.e. fossae not considered), because this is the most suitable parameter, which fits with developmental models (dental size equivalent of bud area; [4]). Reduced major axis (RMA) regressions were used for all linear regressions on the different plots of hypsodont and hypselodont taxa (electronic supplementary material, table S3). This method performed with PAST [35] allows accurate comparison with the regression results of Kavanagh *et al*. [4] to test whether the present regressions follow the IC model, as realized in previous studies in mammals (e.g. [38,39]).

### 2.4. Investigating dental migration in the jaw

**Figure 5.**
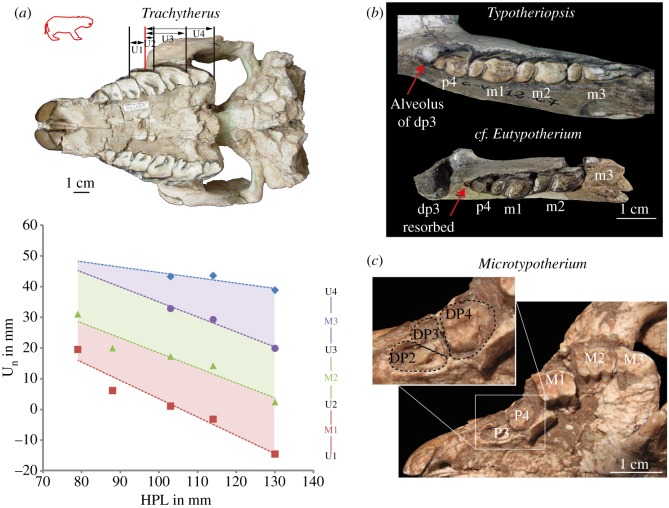
Evidence of dental mesial drift in Mesotheriidae. (*a*) Characterization of upper dental mesial displacement by using cranial measurements on specimens of *Trachytherus alloxus* [40]. The red line represents the reference for measurements (*U*_n_ distances) shown on an adult specimen (MNHN-BOL-V 006355); HPL means hard palate length. (*b*) Mandibular bone remodelling and lower dental resorption are observed in Mesotheriinae (*Typotheriopsis* sp. MLP 76-VI-12-67 and cf. *Eutypotherium lehmannitschei* MLP 91-IX-7-25) and indicated by red arrows. (*c*) Evidence of dental drift indicated by dental eruption of upper permanent premolars in *Microtypotherium choquecotense* (MNHN-BOL-V 003349).

Dental mesial drift (or migration) was reported in Ctenodactylidae, as the consequence of the pressures exerted by the eruption and prolonged growth of distal molars pushing mesial teeth to the front of the jaw [28]. Dental displacement in mesotheriids was estimated by using, for the upper dentition, the anterior part of the zygomatic arch (due to fragmentary material) as the starting point (i.e. spatial reference) and the mesial or distal base of molars as the final points for each measurement (*U*_n_ distances), which are calculated with ImageJ (figure 5). These measurements evaluated the displacement of each molar with respect to the zygomatic arch. They were only measured in *Trachytherus* for which well-preserved skulls were available for juvenile and adult specimens [40]. Nonetheless, these data were complemented by osteological observations on mesotheriines dentitions (*Typotheriopsis* sp. MLP 76-VI-12-67, cf. *Eutypotherium lehmannitschei* MLP 91-IX-7-25, and *Microtypotherium choquecotense* MNHN-BOL-V 003349), and were then compared with those previously obtained in Ctenodactylidae [28] to assess the occurrence of a drift and its relative intensity.

## 3. Results

Hypsodont taxa generally display dental occlusal patterns ranging from very high complexity in juveniles to a more degenerate pattern in adults (especially for M1 and M2) due to wear in both mesotheriids and ctenodactylids (figures 2 and 3). Dental complexity is far less variable in hypselodont taxa, especially in mesotheriids compared with ctenodactylids, because they mainly include adult specimens. In ctenodactylids, *Ctenodactylus* tends to show a more degenerate pattern than juvenile hypsodont taxa (i.e. specimens of *Pectinator*). By contrast, *Felovia* and *Massoutiera* can also show a higher complexity than hypsodont specimens due to stronger enamel infolding, which represent secondary modifications of the enamel outline (e.g. upper molars, figure 3; electronic supplementary material, figure S1). In mesotheriids, hypselodont taxa generally display a complexity as high as juveniles of hypsodont species, meaning that enamel infolding is strong as well. Hypselodont mesotheriids also show a slight increase of the infolding in the third upper and in lower molars, from the oldest known taxa (e.g. *Eotypotherium*, *Altitypotherium*) to the most recent ones (e.g. *Plesiotypotherium*, *Pseudotypotherium*, *Mesotherium*). Significant correlations between dental size (area) and occlusal complexity were found for a few teeth according to the Kendall's τ test (electronic supplementary material, table S2). This is especially the case of the M3 in both families, irrespective of the mode of crown growth. Hypselodont mesotheriids stand out for showing a significant correlation for most teeth (e.g. *Plesiotypotherium*, *Pseudotypotherium* and *Mesotherium* have larger teeth, and a more complex pattern than oldest taxa; electronic supplementary material, table S1). However, and more generally, there is no significant relationship between dental size and complexity, which is exemplified by small hypselodont mesotheriids having molars as complex as *Trachytherus*, which has larger molars, and *Ctenodactylus* having large teeth but a degenerate pattern (figures 2 and 3; electronic supplementary material, table S1).

Regressions of molar area ratios and comparisons of RMA values to the predictive values of the IC model show that upper and lower dentitions of both ctenodactylids and mesotheriids are not significantly different from this model, except for hypsodont mesotheriids (i.e. *Trachytherus*; figure 4; electronic supplementary material, table S3). Interestingly, similar differences can be noticed between plots of hypsodont and hypselodont taxa in both families. In all of the four investigated cases, the intercept values of the regression lines are higher in hypselodont than in hypsodont groups (figure 4; electronic supplementary material, table S3). More precisely, hypsodont species (*Trachytherus* for mesotheriids, and *Pectinator* for ctenodactylids) plot in the right side of both the mean regression line corresponding to the IC model and that corresponding to hypselodont specimens, meaning that ontogenetic trajectories of molar proportions are shifted towards a relatively larger M2 in these taxa. Regarding developmental trends in hypsodont taxa, juveniles show larger M1 and oldest individuals show larger M3, especially in *Trachytherus* which presents a strikingly high ontogenetic variation of molar proportions (figure 4*a*,*b*). In mesotheriids, hypselodont species show much weaker variations of their molar proportions both inter- and intraspecifically relative to their hypsodont counterpart, possibly due to the presence of only a few identified juvenile specimens. This pattern contrasts with the large range of variation found in both hypsodont and hypselodont ctenodactylids, both including juvenile specimens.

The reduction of all *U*_n_ distances between dental loci and the zygomatic arch during growth in *Trachytherus alloxus* (figure 5*a*) clearly evidences a mesial displacement of upper molars from juvenile to adult specimens. If mesial premolars (P1) tend to be expelled from the tooth row in the adult specimens of this species (figure 5*a*), no evidence of dental resorption or alveolar bone remodelling has been noticed in adult specimens. However, bone remodelling can be observed in more derived mesotheriids (*Typotheriopsis* and cf. *Eutypotherium*, figure 5*b*) showing mandibles with empty dp3 alveoli in one specimen, and alveoli closing due to the non-replacement of dp3 which is resorbed. Moreover, p4 seems to be more mesially located and to occupy this new available space. Eruption of only two upper premolars in alveoli which previously probably hosted three deciduous premolars, is observed on both right and left sides in one juvenile specimen of *Microtypotherium*. This also indicates a reduction of the available space, and a putative dental displacement (e.g. P3 located more mesially than DP3, because it is at the previous location of DP2; figure 5*c*).

## 4. Discussion

### 4.1. Variations of enamel complexity patterns as a result of different heterochronic processes

One of the main points of interest concerns the isolated fossae, whose absence in hypselodont mesotheriids, as in ctenodactylids, is compensated by deeper enamel infoldings. It is generally thought that many rodents have teeth with enamel infoldings, whereas ungulates generally show isolated fossae [9,33]. The presence of these fossae can probably constitute a problem because their maintaining is difficult in the case of ungulates developing hypselodonty, and their presence could preclude the development of hypselodonty, as suggested by Janis & Fortelius [9]. In this context, it remains curious that only a few hypselodont ungulates have developed enamel infoldings at the expense of isolated fossae. Our results on mesotheriid notoungulates show that if infoldings are present in juvenile stages of hypsodont taxa and then leave room for isolated fossae with wear in adult stages, only infoldings characterize the occlusal shape in hypselodont taxa. The presence of lingual infoldings in mesotheriids might rely on the initial mesio-distal stretching (i.e. lengthening) of molars, which permits a stronger development of a median lobe combined with transverse crests, as observed in hypsodont taxa ([27,41], figure 3*a*). This is contrary to most ungulates, such as ruminants, having mainly longitudinal crests. These secondary modifications of the dental pattern certainly allow its morphological optimization [33] for a possible development of hypselodonty dentition in these notoungulates, as in ctenodactylids.

It is worth noticing that infoldings are also observed in small (e.g. Interatheriidae) to large notoungulates (e.g. Toxodontidae), which hinder potential developmental explanations related to size concerning their occurrence when compared with other ungulates. Moreover, and as previously noticed in other ungulates [42] and notoungulates [43], enamel complexity is neither tightly correlated with hypsodonty, nor with dental size in both ctenodactylids and mesotheriids. Nonetheless, most molars, especially lower ones, tend to become more complex with increasing size in hypselodont mesotheriids, contrary to what was observed in equids [36]. Conversely, hypselodont ctenodactylids have either more degenerate or slightly more folded patterns relative to their hypsodont counterpart.

As a matter of fact, two strategies are associated with prolonged dental growth in these groups: degenerate pattern in *Ctenodactylus* (i.e. flat wear combined with highly simplified occlusal pattern) or strong enamel infolding in hypselodont mesotheriids and other hypselodont ctenodactylids. Prolonged dental growth corresponds to a heterochronic shift in development, which mainly corresponds to neotenic processes in which dental stem cells are still activated in the cervical loops of the root ([12]; figure 3). However, at the morphogenetic level, it may correspond to two opposite heterochronic processes. It may represent a paedomorphic process because of the conservation of the juvenile pattern during growth allowed by intercuspal loops continuously contributing to development of infoldings at the expense of fossae along the tooth crown, as in mesotheriids. Alternatively, it may correspond to a peramorphic process, because only latter stages are conserved, as observed in the course of ctenodactylid evolution, involving rapid degeneration to the absence of intercuspal loops (figure 3). Interestingly, this latter process occurred in another group of rodent-like notoungulates, the Hegetotheriidae, which present degenerate cheek teeth (e.g. [44]).

### 4.2. Similar constraints of the IC model on upper and lower molars, and consequences of a prolonged growth on molar proportions

The inhibitory cascade model has been tested in many mammals (e.g. [4,37,39,45]), including notoungulates [38], in order to explain the variations of molar relative sizes. This has been accurately studied on lower molars in many mammals, because Kavanagh *et al*. [4] initially tested *in vitro* the inhibitory effect of the growth of the first lower molar on the rest of the lower dentition in laboratory mice. Our analyses confirmed that the IC model is relevant in upper molars as well in both mesotheriids and ctenodactylids, given that results on upper and lower molars display similar tendencies. These results mean that similar underlying developmental mechanisms channel the patterns of both lower and upper molar proportions, and again emphasize the strong integration of these two parts of the dentition [30,31,46].

Interestingly, while most of our linear regressions on molar sizes closely follow the IC model, our results show that hypsodont taxa, especially mesotheriids, slightly deviate from this model. Scrutinized hypsodont taxa indeed have their ontogenetic trajectory shifted towards larger relative sizes of M2. This particular pattern is probably related with dental wear impacting the occlusal surface notably of M1, which decreases during life because of the relatively limited growth of teeth (figures 3 and 4). These variations of molars' dimensions along wear stages were already documented for several hypsodont notoungulates (using mesio-distal length), including mesotheriids [27]. This important aspect, which was not considered in notoungulates by Wilson *et al*. [38], may influence the shift towards larger sizes of M2. In adult individuals of *Trachytherus*, a worn M1 appears much smaller than right after its eruption, and that artificially emphasizes the importance of M2 size, when comparing their relative proportion at different stages. The relative proportions of M1 versus M3 follow the same trend. In this case, the measured molar proportions in a given individual could correspond to an ontogenetical artefact, which may skew the putative inhibitory effects of M1 on subsequent molars at initial time of formation, and the subsequent ontogenetic and structural adjustments (e.g. trade-off between dental growth, lengthening and cranial growth) which allow the whole dentition to fit the space available in the jaw. That does not seem to affect hypselodont taxa, because of their reduced number of cheek teeth. This particular pattern may also explain the large intragroup variation of dental proportions, which is remarkable in *Trachytherus*, less important in Ctenodactylidae, and probably minimized in mesotheriines due to scarcity of juveniles. More generally, slight deviations from the IC model and intraspecific dental size variations can also be variably linked to other ontogenetic factors (i.e. timing of molar eruption), ecological factors, or phylogenetic history, as previously demonstrated for some mammals, such as rodents, primates and carnivores [39,47,48].

### 4.3. Dental migration, loss and reduction influenced by elongated teeth and prolonged growth

The evolutionary histories of mesotheriids and ctenodactylids are characterized by a reduction of the dentition from hypsodont to hypselodont taxa, which is also observed during ontogeny (figure 1). This loss of teeth during ontogeny could be regarded as the normal process of ageing, because of intense dental wear [49]. This is the case for peg-like teeth in *Trachytherus* (e.g. P1), which rapidly leave room for a diastema between mesialmost incisors and remaining cheek teeth. An important displacement of teeth is observed during skull growth in this species which suggests that these losses can also be driven by the pushing effect of the distal molars migrating mesially, which characterizes a drift. This case of mesial drift is reminiscent of that existing in extant ctenodactylid rodents [28]. Resorption or remodelling phenomena have not been observed for teeth or bone to substantiate a putative drift of the dentition in *Trachytherus*. Moreover, dental wear, strong imbrication of teeth as well as length reduction of mesial molar with wear might reduce the effect of a drift, which may preclude resorption and accelerated loss of mesial teeth in this taxon. Consequently, a dental shift, which corresponds to virtual displacement of teeth due to bone apposition during growth, especially at the rear of the jaw [50], cannot be excluded due to the intensity of the (virtual) displacement of molars.

However, more derived mesotheriids show signs of bone remodelling in the mesial part of their dentition, as well as non-replacement of one deciduous premolar per quadrant (figure 1; e.g. *Plesiotypotherium casirense*, ‘*Plesiotypotherium*’ *minus*; [21,51]), as observed in extant ctenodactylids [28]. These characteristics indicate the occurrence of a slight to moderate mesial drift probably combined with a shift of the dentition in mesotheriids, in general [28]. As observed in Ctenodactylidae, this drift is certainly due to the prolonged to continuous growth of molars, which exerts a mechanical pressure on mesial teeth, while also enlarging during initial growth in both hypselodont mesotheriids and ctenodactylids (electronic supplementary material, table S1; [19]). This dynamic mechanism known in some mammals (e.g. [28,49,50]) may explain the loss of mesial premolars during ontogeny in mesotheriids, and may represent a structural prerequisite towards the reduction of the dentition experienced in late diverging mesotheriids.

### 4.4. Evolutionary and functional implications

Both mesotheriids and ctenodactylids underwent a reduction of the dentition during their evolution accompanied with a prolonged to continuous growth of teeth. Our results show these dental modifications were in some extent facilitated by similar ontogenetic variations and structural adjustments in mesotheriids and ctenodactylids. These include (i) secondary modifications of dental crown and occlusal designs, (ii) a transitory adjustment to the high variation of molar proportions in hypsodont forms signalled by a shift towards larger M2 relative to the IC model, and (iii) a progressive increase in height and length of molars leading to drift and loss of mesial teeth.

More generally, the reduction of the dentition combined with increasing molar length and crown height undoubtedly favour masticatory efficiency [9]. In parallel, this also improves the incisor efficiency in these rodent-like mammals, notwithstanding the size differences existing between these mammals. This may primarily represent an adaptation to comminution of tough plants from the low plant cover involving abrasive particles due to increasingly arid environments occurring in some South American areas by the end of Paleogene [17,19,20,52], and in African areas since the Middle Miocene [28,53]. In that context, comminution of this kind of plants could have been improved by two strategies: increasing complexity by means of infoldings forming deep sulci or isolated fossae which enhance the shearing action of enamel edges on molars (e.g. Mesotheriidae; [13,33,34]), or simplified flat dental occlusal surface combined with the ingestion of mineral particles, which may improve grinding (e.g. *Ctenodactylus*; [9,17]). If the specific presence of enamel infoldings in notoungulates remains elusive compared with other ungulates, it could have also been related to their digestive abilities assumed to be less efficient than in many ungulates such as ruminants, and much closer to their extant relatives, the perissodactyls [17]. Consequently, the amount of food intake may have been comparatively higher and the chewing effort greater in notoungulates [17]. This might therefore be the case in large forms with blunt cusps in which infoldings enhance food comminution, especially resistant food with an abrasive component [34], and that may explain why enamel complexity tends to increase in large mesotheriids during their evolution.

Mesotheriids and ctenodactylids evolved a convergent masticatory apparatus in several morphological and dynamic aspects (e.g. bi-modular and high-crowned dentitions with thick cementum, mesial drift). These specializations potentially involve similar biomechanical constraints. Even if dental morphogenetic differences exist between these clades due to their distinct phylogenetic background, our results show that quite similar ontogenetic and structural adjustments occurred in those taxa evolving prolonged to continuous dental growth, presumably in order to prevent dental disorders and ensure a perennial function in constraining environmental contexts (e.g. high amount of abrasive particles deposited on plants in arid environments). This comparative case study on two distant groups now clearly points to further research to scrutinize the existence of such changes in numerous mammals encompassing hypsodont to hypselodont forms. While similar ontogenetic variations are not necessarily expected, their repeated occurrence may underline the existence of important ontogenetic and structural constraints linked to changes in dental growth. These shared features emphasize the crucial need of considering ontogeny in future evolutionary studies on mammals in order to better understand convergent phenomena, and more generally, the balance between the structure, inheritance and function of complex biological systems.

## Supplementary Material

Supp. Fig.1

## Supplementary Material

Supp. Tab.1

## Supplementary Material

Supp. Tab. 2

## Supplementary Material

Supp. Tab. 3

## Data accessibility

Supporting data are accessible in electronic supplementary material, table S1.
