# Supplementary material for: Ontogenetic variations and structural adjustments in mammals evolving prolonged to continuous dental growth
Source: R Soc Open Sci. 2017 Jul 26;4(7):170494. doi: 10.1098/rsos.170494 (PMC5541567; doi:10.1098/rsos.170494)

**Supp Fig. 1 Upper and lower occlusal dental patterns of extant Ctenodactylidae** (modified from Jaeger, 1971)


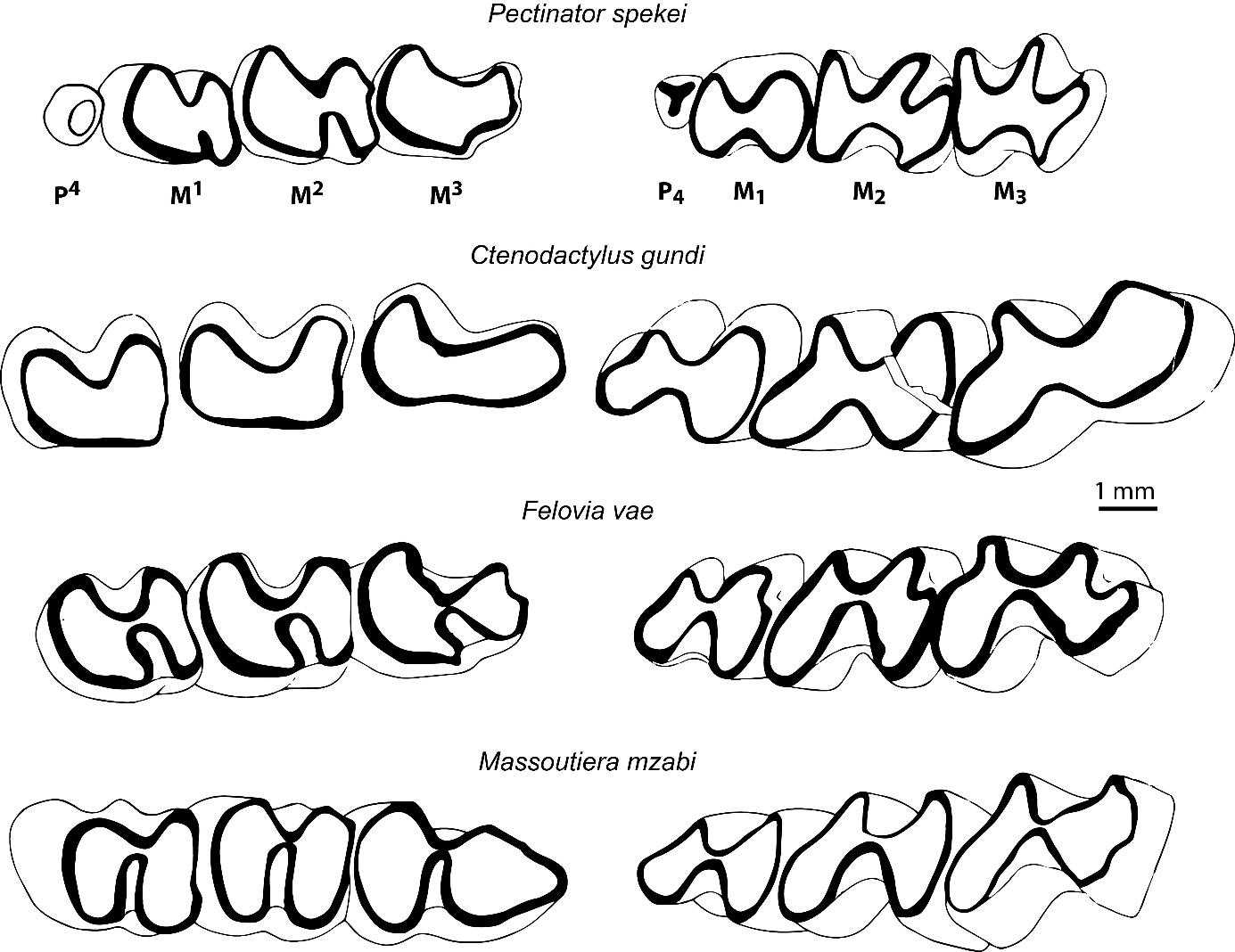

Supplement: Supp. Fig.1 [file rsos170494supp1.docx]
