# Supplementary material for: Ontogenetic variations and structural adjustments in mammals evolving prolonged to continuous dental growth
Source: R Soc Open Sci. 2017 Jul 26;4(7):170494. doi: 10.1098/rsos.170494 (PMC5541567; doi:10.1098/rsos.170494)
Supplement: Supp. Tab. 2 [file rsos170494supp3.doc]

**Ontogenetic variations and structural adjustments in mammals evolving prolonged to continuous dental growth**

Helder GOMES RODRIGUES, Rémi LEFEBVRE, Marcos FERNÁNDEZ-MONESCILLO, Bernardino MAMANI QUISPE, Guillaume BILLET

**Supp. Tab. 2 Test of correlation between dental area and occlusal complexity.** Significance at α=0.05 is indicated in bold.

|  |  | M_1_ | M_2_ | M_3_ | M^1^ | M^2^ | M^3^ |
| --- | --- | --- | --- | --- | --- | --- | --- |
| Hypsodont Mesotheriidae | n | 15 | 12 | 11 | 23 | 23 | 19 |
|  | Kendall's Tau | -0.086 | -0.424 | -0.127 | 0.043 | -0.020 | 0.368 |
|  | p | 0.656 | 0.055 | 0.586 | 0.771 | 0.895 | **0.027** |
| Hypselodont Mesotheriidae | n | 20 | 20 | 18 | 35 | 34 | 34 |
|  | Kendall's Tau | 0.221 | 0.453 | 0.399 | -0.126 | -0.283 | 0.216 |
|  | p | 0.173 | **0.005** | **0.021** | 0.287 | **0.018** | 0.078 |
| Hypsodont Ctenodactylidae | n | 16 | 16 | 15 | 16 | 16 | 15 |
|  | Kendall's Tau | -0.016 | -0.367 | 0.086 | -0.150 | 0.033 | -0.371 |
|  | p | 0.928 | **0.047** | 0.656 | 0.418 | 0.857 | 0.054 |
| Hypselodont Ctenodactylidae | n | 41 | 41 | 39 | 41 | 41 | 39 |
|  | Kendall's Tau | -0.378 | -0.041 | 0.058 | -0.162 | 0.034 | 0.231 |
|  | p | **<0.001** | 0.702 | 0.603 | 0.135 | 0.753 | **0.038** |
