# Supplementary material for: Ontogenetic variations and structural adjustments in mammals evolving prolonged to continuous dental growth
Source: R Soc Open Sci. 2017 Jul 26;4(7):170494. doi: 10.1098/rsos.170494 (PMC5541567; doi:10.1098/rsos.170494)
Supplement: Supp. Tab. 3 [file rsos170494supp4.docx]

**Ontogenetic variations and structural adjustments in mammals evolving prolonged to continuous dental growth**

Helder GOMES RODRIGUES, Rémi LEFEBVRE, Marcos FERNÁNDEZ-MONESCILLO, Bernardino MAMANI QUISPE, Guillaume BILLET

**Supp. Tab. 3 Data relative to reduced major axis analyses showing comparisons of indices between IC model and regressions in hypsodont and hypselodont Mesotheriidae and Ctenodactylidae.** Values of confidence interval (95% CI) for slope and intercept in line with IC model are indicated in bold.

|  |  | n | Slope | 95% CI | Intercept | | 95% CI | r | | p |
| --- | --- | --- | --- | --- | --- | --- | --- | --- | --- | --- |
| IC model | Lower molars |  | 2.000 | - | -1.000 | | - | | 1.000 | - |
| Hypsodont Mesotheriidae | Lower molars | 12 | 2.417 | **1.996; 2.980** | -1.837 | -2.571; -1.131 | | | 0.948 | **<0.001** |
|  | Upper molars | 22 | 2.1165 | **1.574; 2.407** | -1.711 | | -2.101; -1.077 | | 0.962 | **<0.001** |
| Hypselodont Mesotheriidae | Lower molars | 19 | 3.149 | **-5.110; 3.669** | -2.194 | | **-2.819; 6.719** | | 0.0233 | 0.925 |
|  | Upper molars | 34 | 1.566 | **0.951; 2.428** | -0.774 | | **-1.757; -0.065** | | 0.566 | **<0.001** |
| Hypsodont Ctenodactylidae | Lower molars | 17 | 2.075 | **1.600; 2.554** | -1.556 | | **-2.150; -0.940** | | 0.894 | **<0.001** |
|  | Upper molars | 17 | 2.351 | **1.238; 3.094** | -1.834 | | **-2.729; -0.375** | | 0.75 | **<0.001** |
| Hypselodont Ctenodactylidae | Lower molars | 41 | 2.007 | **1.771; 2.573** | -1.245 | | **-2.002; -0.928** | | 0.877 | **<0.001** |
|  | Upper molars | 41 | 2.15 | **1.844; 2.859** | -1.251 | | **-2.005; -0.116** | | 0.839 | **<0.001** |
